# Oxalic Acid Treatment: Short-Term Effects on Enzyme Activities, Vitellogenin Content, and Residual Oxalic Acid Content in House Bees, *Apis mellifera* L.

**DOI:** 10.3390/insects15060409

**Published:** 2024-06-03

**Authors:** Simona Sagona, Elena Tafi, Francesca Coppola, Antonio Nanetti, Chiara Benedetta Boni, Caterina Orlando, Lionella Palego, Laura Betti, Gino Giannaccini, Antonio Felicioli

**Affiliations:** 1 Department of Veterinary Sciences, Pisa University, Viale delle Piagge 2, 56124 Pisa, Italy; simona.sagona@unipi.it (S.S.); francesca.coppola@vet.unipi.it (F.C.); chiarabenedetta.boni3@gmail.com (C.B.B.); 2 Department of Pharmacy, Pisa University, Via Bonanno 6, 56126 Pisa, Italy; caterina.orlando@unipi.it (C.O.); laura.betti@unipi.it (L.B.); gino.giannaccini@unipi.it (G.G.); 3 CREA Research Centre for Agriculture and Environment, Via di Corticella 133, 40128 Bologna, Italy; elena.tafi@crea.gov.it (E.T.); antonio.nanetti@crea.gov.it (A.N.); 4 Department of Clinical and Experimental Medicine, Pisa University, Via Savi 10, 56126 Pisa, Italy; lionella.palego@unipi.it

**Keywords:** oxalic acid, *Apis mellifera*, glucose oxidase, phenoloxidase, glutathione S-transferase, vitellogenin, *Varroa*-treatment

## Abstract

**Simple Summary:**

*Varroa destructor* is a mite that parasitises honeybee hives, weakening colonies and transmitting viruses. Beekeepers apply acaricide treatments to bee colonies to limit the spread of *Varroa* and the resulting negative effects on bee health. Oxalic acid treatment is the most widely applied, but little is known about its potential effects on the physiology of bees, particularly their immune system. This study investigated the short-term effects of oxalic acid treatment on the immune and antioxidant systems of house bees (i.e., glucose oxidase, phenoloxidase, glutathione S-transferase, catalase activities, and vitellogenin content). Residual concentrations of oxalic acid on the bees’ external body and in the haemolymph were also measured. The results showed that the treatment did not affect the concentration of oxalic acid in the haemolymph, in which it was constitutively present. Residues of oxalic acid remained on the outer body of the bees for up to 48 h after treatment. Both glucose oxidase activity and vitellogenin concentration were affected by the treatment, increasing significantly after 48 and 24 h, respectively. No effects were observed on the other parameters investigated (phenoloxidase, glutathione S-transferase, and catalase activities).

**Abstract:**

Honeybees (*Apis mellifera* L.) have to face many challenges, including *Varroa destructor* infestation, associated with viral transmission. Oxalic acid is one of the most common treatments against *Varroa*. Little is known about the physiological effects of oxalic acid, especially those on honeybees’ immune systems. In this study, the short-term effects (0–96 h) of oxalic acid treatment on the immune system components (i.e., glucose oxidase, phenoloxidase, glutathione S-transferase, catalase activities, and vitellogenin contents) of house bees were preliminarily investigated. Oxalic acid contents of bee bodies and haemolymphs were also measured. The results confirm that oxalic acid is constitutively present in bee haemolymphs and its concentration is not affected by treatment. At 6 h after the treatment, a maximum peak of oxalic acid content was detected on bees’ bodies, which gradually decreased after that until physiological levels were reached at 48 h. In the immune system, the oxalic acid treatment determined a peak in glucose oxidase activity at 48 h, indicating a potential defence response and an increase in vitellogenin content at 24 h. No significant changes were recorded in phenoloxidase, glutathione S-transferase, and catalase activities. These results suggest a time-dependent response to oxalic acid, with potential immune system activation in treated bees.

## 1. Introduction

Honeybees (*Apis mellifera* L.) are outstanding pollinators of both wild and cultivated plants and are historically managed in large amounts for pollination purposes and the commercialisation of hive products (e.g., honey, bee pollen, beeswax, propolis, and royal jelly) [1]. In the last few decades, honeybee colonies have faced increasing adversity, including climate change, drought, flowering shortages, exposure to agrochemicals, and a wide range of pests and pathogens [2]. The interaction and synergy of these factors can lead to colony losses [3,4,5]. 

Among pests, the ectoparasitic mite *Varroa destructor* is globally one of the main threats to honeybees [6]. The *Varroa* life cycle is closely linked to honeybee population dynamics [7]. Female mites enter uncapped brood cells, where they lay eggs and mate [7]. When the host bee emerges from its cell, the adult female mites exit with her and enter a phoretic phase of spreading in the hive, during which, they feed on bees’ haemolymphs and fat bodies [8]. *Varroa* causes direct damage to bees, altering their physiology, behaviour, and haemolymph composition and leading to body weight loss due to their feeding activity [9,10,11]. However, the worst effects of *Varroa* infestation are due to the transmission of associated viruses that can replicate in the mite (e.g., the deformed wing virus and the AKI-complex viruses) and lead to colony mortality [12,13]. 

Therefore, beekeepers and the scientific community have put considerable effort into the research and development of mechanical and chemical control methods to limit the spread of *Varroa* [8]. Mechanical methods consist of brood removal or queen caging to ensure a broodless period [14]. Chemical control relies on the application of synthetic and organic acaricides [14]. The latter is increasingly being used by beekeepers to avoid the adverse effects that synthetic compounds have on both bees and bee products and the onset of resistance [15,16,17]. The most common natural compounds are essential oils, mainly thymol, and organic acids, such as formic and, particularly, oxalic acid [18]. Oxalic acid is naturally present in honey in a concentration range of 3.3–771.4 mg/kg, and, due to its hydrophilic nature, no residue is left in propolis or beeswax [19,20]. Oxalic acid is generally well tolerated by adult bees in concentrations of up to 4.6% [21,22,23], while it is toxic for honeybee larvae even at low concentrations (<1%) [24]. Acaricide treatment with oxalic acid is usually carried out by spraying or trickling the acid dissolved in a sugar solution [14]. The administration of oxalate crystals by sublimation is also possible [21,25,26]. In the presence of a brood, the application of oxalic acid is usually performed in combination with queen caging to create a broodless period that ensures the exclusive presence of phoretic mites on adult bees that are easily accessible to the acaricide [20,27]. 

The adverse effects of oxalic acid on bees include damage to the gut by topical or oral administration, an increase in mortality under laboratory conditions, and the reduction of the brood in the colony. However, the physiological effects are not fully known, especially at the immune level [28,29,30]. Immune system components that may be affected by oxalic acid treatment include glucose oxidase, phenoloxidase, and vitellogenin [31]. The enzyme glucose oxidase is part of the bee’s social immune system, together with hygienic behaviour, and is involved in the conversion of glucose in gluconic acid and hydrogen peroxide, with antimicrobial activity [32,33]. Phenoloxidase is an enzyme of a bee’s individual innate immune system involved in the encapsulation of pathogens and nodule formation through the production of melanin [34]. Vitellogenin also plays a role in individual immunity, providing hemocytes with the zinc required for their immune function, and is involved in ageing regulation [35,36]. Oxalic acid effects could also involve enzymes of the antioxidant system such as glutathione S-transferase and catalase [37,38].

Variations in vitellogenin content (i.e., a reduction in newly emerging bees and an increase in nurses) and glucose oxidase activity (i.e., a reduction in drones) in the first bee generation after oxalic acid treatment combined with queen caging have recently been highlighted by Sagona et al. [31]. Based on these findings, this investigation aimed to deeply understand the effects of oxalic acid on adult bees directly subjected to the common acaricide treatment applied by beekeepers. With this aim, the short-term (0, 6, 24, 48, and 96 h) effects of the treatment on the welfare status of house bees were investigated by analysing the activity of the enzymes of the honeybee immune system (i.e., glucose oxidase and phenoloxidase), the vitellogenin content, and the activity of two antioxidant enzymes (i.e., glutathione S-transferase and catalase) and measuring the residual content of oxalic acid in the haemolymphs and on the bees’ bodies.

## 2. Material and Methods

### 2.1. Sampling and Haemolymph Collection

House honeybees were collected from the apiary of CREA Bologna (44°52′43.94″ N–11°34′93.76″ E) from a hive previously managed with queen caging to obtain a strong family (adult/brood) in the absence of the principal honeybee’s disease symptoms (i.e., American foulbrood, deformed wings, diarrhoea). Worker bees present on internal frames, except for newly emerged bees, were sampled as house bees. 

In July 2022, a pool of 200 house bees were randomly collected from the same hive, of which 100 (i.e., 20 bees/interval time) were collected before oxalic acid treatment (PRE bees) and 100 (i.e., 20 bees/interval time) were collected after treatment (POST bees). PRE bees were sampled at the following time intervals: at time 0 (T0) and after 6 (T6), 24 (T24), 48 (T48), and 96 (T96) hours. POST bees were sampled at the following time intervals: at 6 (T6), 24 (T24), 48 (T48), and 96 (T96) hours after oxalic acid treatment. The POST T0 time interval was included in the PRE group because they were untreated bees. The PRE group was the control group. For each time interval for both PRE and POST groups, 24 bees (i.e., 6 pools each contained 4 bees) were also sampled for haemolymph collection. The oxalic acid treatment consisted of a dose of 50 mL/hive with Api-Bioxal (Chemicals laif, Padua, Italy) (i.e., oxalic acid dihydrate 62 mg/mL).

Sampled bees were anesthetised by freezing, and 3 µL of haemolymph per bee was withdrawn from the thorax by the insertion of a 1 μL glass microcapillary through the neck membrane. The haemolymph was collected in pools of 4 bees and stored in PBS (80 µL of PBS × 12 µL of haemolymph) at −20 °C. 

Spectrophotometric/colourimetric analyses were performed by a Multiskan FC reader (Thermo Scientific, Waltham, MA, USA) and a Lambda 25 UV/VIS spectrometer (PerkinElmer, Waltham, MA, USA). All chemicals were purchased from Sigma-Aldrich (St. Louis, MO, USA).

### 2.2. Oxalic Acid Content

Oxalic acid content was quantified directly from both the external body (body wash) and haemolymph. For oxalic acid quantification from body wash, 18 bees from the control group and 3 from each treated group at different collection times were used (i.e., each bee was a replicate). Each honeybee was washed with 250 µL of distilled water and mixed by vortex for 2 min. The resultant body wash water was used for the determination of oxalic acid concentration. For haemolymph, oxalic acid concentration was measured in 18 pools for the control group and 3 pools for each treated group at different collection times. Oxalic acid content quantification was performed by using the oxalic acid colourimetric assay kit from Sigma-Aldrich (catalogue number MAK179) according to the manufacturer’s instructions. Oxalic acid concentration was determined by a coupled enzyme reaction, which resulted in a colourimetric product proportional to the oxalate present, recorded at 450 nm. Values were expressed as nmol/µL.

### 2.3. Enzymatic Assays

For enzymatic assays, 36 bees (i.e., each bee used as a replicate) for the control group and 6 for each treated group at different collection times were used. Glucose oxidase determination was carried out on protein extracts from honeybee heads. Each head was weighed and crushed with a Teflon pestle in 150 µL of 100 mM phosphate buffer (pH 7.2) with 1% (*v*/*v*) Triton X-100. The supernatant of each sample was collected after decantation. The pellets were incubated in 150 µL of 100 mM phosphate buffer (pH 7.2) and allowed to decant again. The second supernatants were collected and mixed with the first ones, and their total protein concentration was measured by a Qubit 2.0 fluorimeter (Invitrogen, Waltham, MA, USA). For glucose oxidase activity measurement, a solution of 100 mM Hepes buffer (pH 7.0), 0.1 mM EDTA, and 5 mM D-glucose was first added to the samples [39]. Before the spectrophotometer reading, 0.18 mg/mL diaminobenzidine (DAB) and 0.02 mg/mL horseradish peroxidase (HRP) were also added to the samples. Absorbance was then measured at λ = 352 nm at time 0 and after 120 min. The resulting values were expressed as U/mg of protein [39].

Phenoloxidase, glutathione S-transferase, and catalase activities were measured on protein extracts of bee thoraxes [33]. Each sample was weighed before protein extraction and 200 µL of 100 mM phosphate buffer, pH 7.2, with 1% (*v*/*v*) Triton X-100 was added. Samples were homogenised by a Teflon pestle and allowed to decant. The resulting supernatants were collected, while 200 µL of 100 mM phosphate buffer, pH 7.2, was added to pellets and allowed to decant. The supernatants were mixed with those previously collected and the total protein concentration was measured by a Qubit 2.0 fluorimeter (Invitrogen, CA, USA). 

For the phenoloxidase activity assay, 7 µL of the sample was loaded onto a 96-well plate with 63 µL of phosphate saline buffer, pH 7.4, and 90 µL of milliQ water, in accordance with Mazzei and colleagues [40], with some modifications. The 96-well plate was incubated at 37 °C for 5 min in a PST-60HL thermos shaker (Biosan, Riga, Latvia) and 40 µL of L-3,4-dihydroxyphenylalanine (L-dopa) (2 mg/mL) was then added. Absorbance data were obtained at λ = 490 nm at times 0, 5, 10, and 15 min. Values were expressed as U/mg of protein.

For glutathione S-transferase activity measurement, a solution made of 150 µL of 100 mM phosphate buffer (pH 6.5), 6.5 µL of 1 mM 1-chloro-2,4-dinitrobenzene (CDNB) in methanol, 25 µL of distilled water, and 12 µL of 5 mM GSH were incubated at 30 °C for 5 min, according to a slightly modified method from Habig et al. [41]. Then, 6.5 µL of each thorax protein extract was added to the solution and absorbance was measured at λ = 340 nm at 0, 5, and 10 min. The resulting values were expressed as U/mg of protein.

Catalase activity was analysed using the method by Góth [42]. Briefly, samples were incubated with 1 mL of 65µM H_2_O_2_ in 60 mM PBS, pH 7.4, for 60 s. Two control reactions were prepared with H_2_O_2_ in 60 mM PBS, pH 7.4 (no enzyme control), and only 60 mM PBS, pH 7.4 (no enzyme/no substrate). The reaction was stopped by adding 32.4 mM ammonium molybdate to the samples and control reactions. The absorbance was determined at 405 nm by the yellow molybdate and H_2_O_2_ complex against the no enzyme/no substrate blank. Values were expressed as U/mg of protein. 

### 2.4. Vitellogenin Content

Vitellogenin content was measured for 40 µL of each haemolymph pool sample for both the control and each treated group at different collection times diluted 1:2 by a General Vitellogenin ELISA Kit (catalogue number 0772-E0010Ge, Bioassay technology laboratory, Shanghai, China). Haemolymph samples were added to kit plates pre-coated with general VG (vitellogenin) antibody, allowing the vitellogenin contained in the samples to bind to the antibody. In the wells of the plate, a biotinylated general VG antibody was then added, which bound to the samples. The biotinylated VG antibody in turn bound to the streptavidin-HRP that was subsequently added to the samples. A washing step was carried out after incubation to remove the unbound streptavidin-HRP from the plate. Lastly, a substrate solution was added to allow for colour development in proportion to the amount of general VG antibody that was bound. An acidic stop solution ended the reaction, and the absorbance of the samples was then measured at 450 nm.

The mean blank value was deducted from the data obtained and then fitted to the calibration curve (obtained with the standards) using the MyCurveFit.com program, obtaining the corresponding µg vitellogenin/µg of protein for each sample. 

### 2.5. Statistical Analysis

Data were statistically processed using JMP software 7 (SAS Institute, 2008). Data regarding untreated bees (control group) were processed as a single group to limit non-physiological fluctuations due to external or internal factors. All enzymatic activities and both oxalic acid and vitellogenin contents were processed as follows. After checking that the data were not normally distributed using the Shapiro–Wilk test, they were processed using the non-parametric Wilcoxon test. Data were also tested for homogeneity of variances using the Bartlett test. In all analysed parameters, differences among treatments were assessed by using the non-parametric Kruskal–Wallis H-test, followed by post hoc Mann–Whitney U-test pairwise comparisons. Differences with *p* < 0.05 were considered statistically significant.

## 3. Results

### 3.1. Oxalic Acid Content

**Figure 1:**
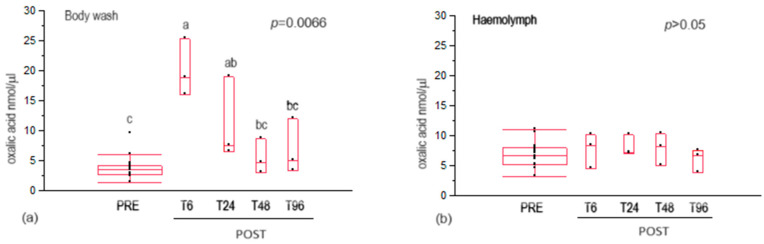
Residual oxalic acid contents in the house bee body wash (**a**) and haemolymph (**b**). Data are expressed as nmol/µL of oxalic acid. T6, T24, T48, T96 = time in hours after the treatment at which the bees were collected (POST bees); PRE = bees collected before the oxalic acid treatment. Different letters above the plots indicate statistically significant values for *p* < 0.05.

The residual of oxalic acid content in the body wash was significantly higher at 6 h after treatment than in control bees (PRE bees), (Figure 1a). A decrease in oxalic acid content was recorded from T6 to T48 post-treatment, with a significant reduction at T48 compared to T6.

In the haemolymphs of house bees, no significant variation was detected in oxalic acid content among different collection times (Figure 1b). 

### 3.2. Enzymatic Assays

**Figure 2:**
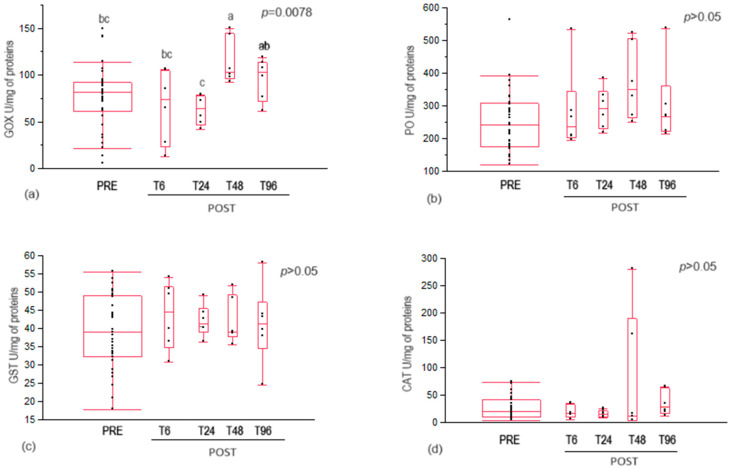
(**a**) Glucose oxidase activity, (**b**) phenoloxidase activity, (**c**) glutathione S transferase activity, and (**d**) catalase activity in control (PRE) and treated (POST) house bees with acid oxalic. T6, T24, T48, T96 = time in hours after treatment at which the bees were collected. Different letters above the plots indicate statistically significant values for *p* < 0.01.

Glucose oxidase activity significantly increased in bees at 48 h after oxalic acid treatment, reaching a minimum peak at 24 h post-treatment (Figure 2a). 

No statistical differences were recorded in phenoloxidase, glutathione S-transferase, and catalase activities between control (PRE) and treated (POST) bees at different collection times (Figure 2b–d). 

### 3.3. Vitellogenin Content

**Figure 3:**
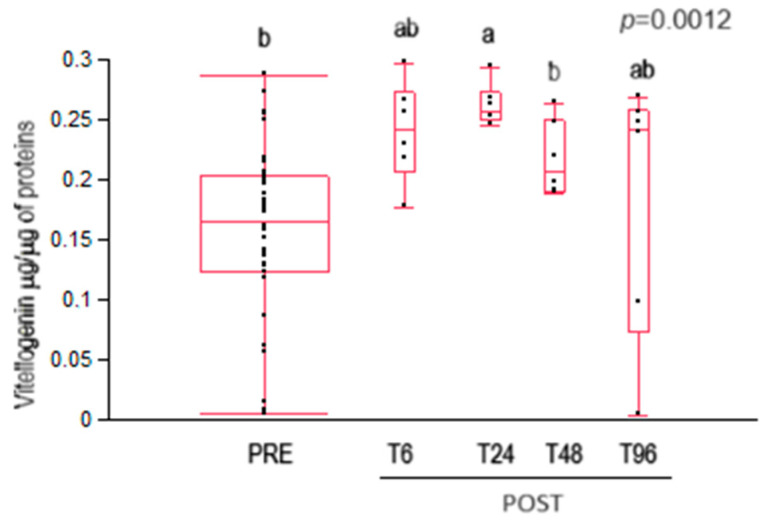
Vitellogenin content in untreated (PRE) and treated (POST) house bees with acid oxalic. Data are expressed as µg of vitellogenin/µg of protein. T6, T24, T48, T96 = time in hours after treatment at which the bees were collected. Different letters above the plots indicate statistically significant values for *p* < 0.01.

The vitellogenin content was significantly higher 24 h after oxalic acid treatment compared to control bees (PRE) (Figure 3). A decrease in vitellogenin content was recorded in treated bees from T24 to T48.

## 4. Discussion

The results obtained in this investigation suggest a time-dependent response of house bees to oxalic acid treatment, with a potential immune system activation in treated bees.

### 4.1. Oxalic Acid Content

The residual oxalic acid content in the honeybee body wash and the hemolymph followed a different trend. In the body wash of treated bees, the residual oxalic acid content increased, with a peak at 6 h post-treatment, returning to the same as that of untreated bees (control) after 48 h. This indicates that the oxalic acid contained in the treatment solution could persist on the bees’ cuticles for at least 24 h. The bees’ self-grooming behaviour, which was observed to increase under oxalic acid treatment [43], may have resulted in the removal of oxalic acid crystals deposited on their bodies, contributing to the progressive decrease in residual oxalic acid content in the body wash. This result confirms what was previously observed by Nanetti et al. [44], who reported maximum bee contamination 24 h after oxalic acid treatment, with a decrease in the following hours. The differences recorded in the time of the maximum peak of oxalic acid contamination recorded in this work compared to that of Nanetti et al. [44] could be due to different methods of oxalic acid content determination (i.e., radioactivity measurement *versus* colourimetric method) and the different matrix analysed (i.e., whole bee *versus* bee body wash). No data are yet available on the trend of oxalic acid content between both 0–6 and 6–24 h, and further investigations sampling bees at closer time intervals are desirable. 

In the haemolymph of house bees, no significant variation in oxalic acid content was detected for the different collection times. It is well known that oxalic acid is one of the products of some metabolic cycles (e.g., Krebs cycle) [45]. Oxalic acid is also usually synthesised by the bee’s organism, making it one of the most abundant acids in honey, ranging from 11 to 119 mg/kg [46]. To the best of our knowledge, only Nozal et al. [47] reported the presence of oxalic acid in honeybee haemolymph, at a concentration of 0.06 µg/bee. The results obtained in this study confirm that oxalic acid is constitutively present in the haemolymph of the honeybee and the oxalic acid treatment did not affect its concentration in the investigated time intervals. On the other hand, Nanetti and colleagues [44] reported a peak of oxalic acid content in the haemolymphs of bees 12 h after treatment, and a subsequent decrease to a minimum level after 84 h. Since the 12 h sampling time interval was not investigated in this study, the presence of a possible peak cannot be excluded. Furthermore, in the experiments performed by Nanetti et al. [44], oxalic acid was administrated in combination with 60% sucrose syrup, while, in this work, oxalic acid was combined with glycerol. Although both sucrose and glycerol have a hygroscopic function, a higher ingestion of oxalic acid by honeybees was observed when the treatment was administered in a sugar solution compared to a sugar-free formulation [48,49]. Therefore, it can be speculated that the peak of oxalic acid reported by Nanetti et al. [44] could have resulted from the high ingestion of oxalic acid as a combined consequence of usual bee hygienic behaviour and the presence of sugar.

### 4.2. Immune System Enzymes

Concerning the honeybee immune system, oxalic acid treatment determined a significant increase in glucose oxidase activity at 48 h after oxalic acid administration. At this time, a significant decrease in the oxalic acid content of bee body wash was also recorded. Therefore, since glucose oxidase is part of the bee’s social immune system, the increase in its activity could be a prompt defence response to the presence of this foreign, external chemical compound [32,33]. This hypothesis can be confirmed by the absence of effects on the glucose oxidase activity of second-generation nurse bees after oxalic acid treatment recorded by Sagona et al. [31], probably due to the absence of direct exposure to the chemical compound functioning as an activator stimulus of the social immune system.

No statistical differences were recorded in phenoloxidase activity at different collection times.

Phenoloxidase is a constitutive enzyme in bees, but it can also be induced under certain conditions [34]. An increase in phenoloxidase activity in response to stress is linked to a non-decrease in bee survival [39]. Although bee survival was not measured, given the lack of activation of phenoloxidase activity, it can be assumed that oxalic acid treatment did not induce individual immune stress in the bees.

### 4.3. Antioxidant Enzymes

No statistical differences were recorded in antioxidant enzymes, glutathione S-transferase, and catalase activities at different collection times. This result agrees with those of Rouibi and colleagues [50], who observed no significant changes in glutathione S-transferase activity over time in adult bees, including nurses, treated with a 3% oxalic acid solution [50]. Sagona et al. [31] also observed that oxalic acid treatment had no significant effect on catalase activity in bees belonging to the generation following the one that had received the treatment. The activity of catalase can function as a marker of honeybee physiological conditions and represents the primary defence against reactive oxidative species [51]. As no variations in the activity of this enzyme were observed, it can be supposed that oxalic acid treatment did not significantly increase the honeybees’ oxidative stress and had no significant detrimental effects on bee welfare/physiology. A study by Çalişkan [45] suggested the existence of an oxalate oxidase pathway that produces hydrogen peroxide from oxalate. Therefore, further investigations of catalase, which acts just on hydrogen peroxide, would be advisable.

### 4.4. Vitellogenin

Oxalic acid treatment induced an increase in vitellogenin content after 24 h, which decreased again after 48 h. Cabbri and colleagues [52] also observed an increase in vitellogenin content in worker bees from colonies treated with oxalic acid. Moreover, these results are consistent with those of Sagona et al. [31], who observed that the vitellogenin content was higher in nurse bees belonging to the generation following the one treated with oxalic acid than the untreated ones. Since the vitellogenin content changed in the haemolymph, despite no variation in the content of oxalic acid, it cannot be excluded that oxalic acid content changes and promotes vitellogenin content variations at time intervals not considered in this investigation. Furthermore, Nozal and colleagues [47] observed that by treating bees topically with oxalic acid, oxalic acid was also detected within some organs of the bee, suggesting the permeation of oxalic acid through the cuticle. Thus, further investigations of vitellogenin and oxalic acid contents in the haemolymph are desirable.

## 5. Conclusions

The results obtained in this investigation confirm what was reported by Nozal and colleagues [47], that oxalic acid is constitutively present in bee haemolymphs. Within the time intervals investigated in this work, the haemolymph oxalic acid concentration was not affected by oxalic acid treatment. 

At 24 h after treatment, oxalic acid content on the bee body wash decreased until physiological levels were reached after 48 h. At 24 h and 48 h after treatment, peaks in vitellogenin content and glucose oxidase activity were respectively recorded. The results suggest a prompt defence response to the presence of this foreign, external chemical compound, but further investigations of potential changes that occur between 24 and 48 h post-treatment are desirable to better understand the effects of oxalic acid on honeybee immune responses. Furthermore, further understanding the molecular mechanisms in which vitellogenin and oxalic acid are involved would be desirable. Since oxalic acid did not seem to affect the activity of the investigated antioxidant enzymes (catalase and glutathione S-transferase), this treatment appeared to have no negative effects on the antioxidant capacity of the treated bees. Furthermore, the lack of an effect of oxalic acid treatment on phenoloxidase activity could be a positive factor. In conclusion, oxalic acid seems to be a good tool for *Varroa* control as it appears to have no negative impact on bee welfare. Following oxalic acid treatment, it would also be interesting to investigate the possible enrichment by honeybees of its content in the honey.

## Data Availability

The raw data supporting the conclusions of this article will be made available by the authors upon request.
